# Identification and Association of *CYP2R1, CYP27B1*, and *GC* Gene Polymorphisms with Vitamin D Deficiency in Apparently Healthy Population and in Silico Analysis of the Binding Pocket of Vitamin D3

**DOI:** 10.3390/cimb47100849

**Published:** 2025-10-15

**Authors:** Saima Manzoor, Asifa Majeed, Palvasha Waheed, Amir Rashid

**Affiliations:** Department of Biochemistry and Molecular Biology, Army Medical College, National University of Medical Sciences, Abid Majeed Road, Rawalpindi 46000, Pakistan; p_waheed@hotmail.com (P.W.); dramiramc@yahoo.com (A.R.)

**Keywords:** vitamin D deficiency, 25 alpha-hydroxylase, tetra ARMS PCR, sequencing, computational modeling

## Abstract

Vitamin D deficiency is highly prevalent in Pakistan, but there is limited data on its genetic aspects. This case–control pilot study aimed to determine the association of rs782153744, rs200183599, rs118204011, and rs28934604 with vitamin D deficiency along rs7041 which has been studied in our population. The DNA of a total of 600 subjects (300 cases and 300 controls) was extracted and genotyped by tetra ARMS PCR, followed by Sanger DNA sequencing of exon 4 of the *CYP2R1* and *CYP27B1* genes and exon 8 of the *GC* gene. SNP Stat was employed to analyze the data, while logistic regression was used to calculate the *p*-values and odds ratios (ORs). The R package version R studio (2025.05.1) Build 513 was used to statistically analyze rs782153744. In silico modeling of wild and mutant *CYP2R1* and *GC* proteins was performed in Swiss-Model, Swiss-Dock, Discovery Studio, and PyMol using 3c6g and IJ78 as templates to perform binding pocket analysis of vitamin D3. The rs782153744 showed a protective association in the additive (OR: 0.15, 95% CI: 0.08–0.27, *p*-value < 0.001), recessive (OR: 0.19, 95% CI: 0.10–0.33, *p*-value < 0.001), and dominant (OR: 0.19, CI = 0.10–0.33, *p*-value < 0.001) models, while *GC* rs7041 (T > A, T > G) displayed a *p*-value < 0.0001 across all genetic models. Sanger sequencing yielded insignificant results, and the SNPs rs200183599, rs118204011, and rs28934604 had no significant association with vitamin D deficiency. The molecular pocket analysis of wild and mutant *CYP2R1* proteins carrying rs782153744 polymorphisms revealed no changes. *GC* proteins carrying the rs7041 polymorphism revealed a shift in their 3D and 2D configuration, as well as a change in the amino acid residue of the binding pocket of VD3. The risk-associated rs7041 and protective rs782153744 variants back genetic screening for vitamin D deficiency risk stratification, allowing targeted supplementation in predisposed subjects and assisting in formulating a genotype-specific therapeutic approach.

## 1. Introduction

Sunlight is the primary source of vitamin D in the skin. Two hydroxylation reactions lead to vitamin D’s activation, with the first carried out in the liver by the enzyme 25 alpha-hydroxylase encoded by the *CYP2R1* gene, and the second carried out in the kidneys by one alpha-hydroxylase encoded by *CYP27B1* [1]. The most crucial purpose of vitamin D is to regulate calcium and phosphorus levels in the body. It keeps them at normal levels through its effects on the kidneys, intestines, and bones, with a nuclear vitamin D receptor protein (*VDR*) mediating most of its effects, and it is present in many cells to regulate gene expression [2]. Research has linked vitamin D deficiency (VDD) with the onset of other significant diseases, such as breast cancer [3], type 2 diabetes [4], cardiovascular disease [5], and depression [6]. More than 70 studies have reported an association of vitamin D deficiency with tuberculosis, cardiovascular diseases, diabetes, cancer, and problems related to periodontics, bones, and the musculoskeletal system in the Pakistani population [7].

The economic efficiency of treating VDD enables individuals to live healthy lives and reduces hospital expenses [8]. An observational, cross-sectional study was conducted on South Asians living in the UK (N = 6433), and it revealed exceptionally low levels of serum vitamin D in this population [9]. A single laboratory-based study conducted on 60,937 samples from all provinces of Pakistan reported that VDD was prevalent in 66.1% of the population [10]. In humans, it was found that genetic aspects may affect vitamin D bioavailability in the circulation by approximately 53% to 68.9% [11]. A case–control study was conducted in China (506 Han children) to observe whether there is an association between three vitamin D pathway genes and the risk of rickets. The 12 polymorphisms of the *CYP2R1, GC,* and *DHCR7/NADSYN1* genes were genotyped using the SNPshot assay technique, and five SNPs showed significant associations with rickets under three different genetic models [12]. In a systematic review, *CYP2R1* gene polymorphisms (rs10766197, rs2060793, and rs10741657) and *GC* gene polymorphisms (rs7041, rs4588, rs2282679, and rs1155563) were confirmed to have an association with serum vitamin D levels in more than 50% of studies [13]. Computational modeling of vitamin D metabolism genes established that many polymorphisms affect proteins involved in signaling, activation, and inactivation of vitamin D, and the authors concluded that multiple-gene analysis of vitamin D metabolism is key to understanding the phenotypic variations related to low levels of vitamin D [14].

There is a significant gap in the research on vitamin D gene polymorphisms in Pakistan. Local studies discuss biochemical parameters and single gene variant without in silico modeling. Local research has reported the relation of *CYP2R1* with tuberculosis [15], *CYP27B1* with polycystic ovarian syndrome [16], the *GC* polymorphism with VDD (rs7041, rs4588), *GC* with asthma [17], *GC* with type 2 diabetes [18], and *GC* with acute myocardial infarction [19], while a single study reported that *CYP2R1* polymorphisms (rs10766197 and rs10741657), *VDR* polymorphisms (rs2228570 and rs7975232), and *CYP27B1* genetic variants (rs10877012) are associated with VDD and conditions such as hypertension and diabetes mellitus [20].

Polymorphisms can influence an individual’s vulnerability to ailments like osteoporosis, cardiovascular diseases, depression, and cancer. Identifying and associating these polymorphisms would allow us to predict disease risk and adopt preventive measures. Moreover, awareness of polymorphisms would educate people about the relationship between genetics and health and enable them to make informed decisions to maintain good health.

In this research, multiple vitamin D metabolism genes were selected to identify the genetic variants that may be involved in VDD. The rs7041 (the *GC* gene) has been studied extensively in various populations. However, this pilot study aims to investigate rs200183599, rs782153744 (*CYP2R1),* rs28934604, and rs118204011 *(CYP27B1)* polymorphisms, which have never been studied in the Pakistani population. Moreover, the SNP database shows no publications about rs782153744.

## 2. Materials and Methods

### 2.1. Study Population

This study was conducted in the Biochemistry and Molecular Biology department of the Army Medical College, National University of Medical Sciences (NUMS), from December 2020 to December 2024. The sample size was calculated with the WHO sample size online calculator (https://cdn.who.int/media/docs/default-source/ncds/ncd-surveillance/steps/sample-size-calculator.xls, accessed on 4 July 2020). Vitamin D deficiency was reported to have a prevalence of 59.9% [21]. Of the 600 participants enrolled in the study, the calculated sample size was 369.

A pilot study was carried out to analyze the rs10741657 polymorphism of the *CYP2R1* gene in 30 cases with low vitamin D levels and 10 controls with sufficient vitamin D levels [22]. This is also a pilot study that was conducted to explore the role of the variants rs200183599, rs782153744, rs118204011, and rs28934604 in relation to VDD in apparently healthy members of the Pakistani population. Seven hundred fifty-two apparently healthy subjects were recruited from the Combined Military Hospital using a non-probability purposive sampling technique. Based on vitamin D levels, subjects were divided into three hundred cases and three hundred healthy controls. The operational definition of VDD was <20 ng/mL (<50 nmol/L), while insufficiency was defined as a value of 20–30 ng/mL (50–75 nmol/L), and sufficiency was defined as a value of 30–50 ng/mL (75–125 nmol/L) [23].

Serum vitamin D was measured by an antibody competitive (chemiluminescence method) immunoassay using an ADVIA Centaur (SIEMENS, Munich, Germany). Sanger sequencing of exon 4 of *CYP2R1, CYP27B1*, and exon 8 of the *GC* gene was performed on gender- and age-matched vitamin D-deficient (case) and healthy (control) subjects. The research was conducted according to ethical criteria established by the Declaration of Helsinki [24], and the subjects signed a written informed consent form for the collection of blood samples. The inclusion criteria were apparently healthy individuals screened for vitamin D levels, aged between 20 and 70 years, and of any ethnicity. The exclusion criteria included cases of osteoporosis, obesity, cancer, diabetes, polycystic ovary syndrome (PCOS), or multiple sclerosis; those with immunological, neoplastic, endocrine, hepatic, renal, gastrointestinal, hematological, neurological, or psychiatric diseases; pregnant and lactating mothers; and subjects on vitamin D supplements or with vitamin D toxicity. Both groups were composed of 20% male subjects and 80% female subjects. The average age was 44.98 ± 15.24 in the cases and 43.99 ± 15.30 in the controls.

### 2.2. DNA Isolation

DNA extraction was performed using the phenol–chloroform method [25]. DNA was then quantitatively and qualitatively analyzed using agarose gel electrophoresis. Both procedures’ details are mentioned in the Supplementary Materials.

### 2.3. Selection of Genetic Variants

The SNPs were selected from two databases (https://www.ncbi.nlm.nih.gov, accessed on 26 July 2020). The rs7041 polymorphism of the *GC* gene was studied, as this variant has been reported in the literature to represent a risk factor in vitamin D deficiency [26]. rs200183599, rs782153744 (*CYP2R1)*, rs28934604, and rs118204011 *(CYP27B1*) were also included in the study, as these variants have never been genetically studied in the Pakistani population. Primers of these SNPs were designed through the online software primer 1 (https://primer1.soton.ac.uk/primer1.html, accessed on 6 December 2021) [27], and are given in the Supplementary Materials. The tetra-ARMS polymerase chain reaction (PCR) was used to genotype the single-nucleotide polymorphisms in *CYP2R1, CYP27B1,* and the *GC* gene. The tetra ARMS PCR technique and genotyping of all genetic variants are given in the Supplementary Materials.

### 2.4. Genetic Screening of Exons

Exon 4 of the *CYP2R1* gene, exon 4 of *CYP27B1,* and exon 8 of the *GC* gene were selected for sequencing, and Primer 3 plus software was used to obtain their primers (https://www.primer3plus.com, accessed on 10 November 2022). Primers were validated using the National Center for Biotechnology Information’s Primer Basic Local Alignment Search Tool (NCBI Primer-BLAST, accessible at https://www.ncbi.nlm.nih.gov/tools/primer-blast/, accessed on 29 December 2023). The 5′-3′ sequence of exon 4 of the *CYP2R1* forward primer was TGGCTTGTTATAGGTTATCAGAG, while the reverse primer was TCTCCTGTTAGAATCAGTTCTGTG, and the product size of the *CYP2R1* exon 4 primers was 685bp. The 5′-3′ sequences of exon 4 of the *CYP27B1* forward and reverse primers were CTACCAGAGCCTCCCGGAA and AATACCTCGCTACCCCTGGA, and the product length was 308bp. The 5′-3′ sequence of exon 8 of the *GC* gene forward primer was *TTGCACTTAATAGCCTTATAG,* and the reverse primer was GAAATGAGTGATAGCATACCT, and the product size was 569 bp. The reagents used in the PCR of 3 exons included 17 µL of nuclease-free water, 2.5 µL of Taq buffer (Bio Basic, Toronto, ON, Canada) 1xPCR, 1.5 µL of 25 mM MgCl_2_ (Bio Basic, Canada), 0.5 µL of 10 mM dNTPs (dinucleotide triphosphates) Mix (Bio Basic, Canada}, 1 pmol/µL forward and reverse primers (Macrogen, 238, Teheran-ro, Gangnam-gu, Seoul, Republic of Korea), and 0.5 µL Taq polymerase (Bio Basic, Canada). Each reaction was carried out with 1 µL of genomic DNA. The PCR for *CYP27B1* exon 4 started at a hot temperature of 95 °C for 5 min, and the sample underwent denaturation at 95 °C for 30 s, annealing at 59 °C for 30 s, extension at 72 °C for 30 s, and final extension at 72 °C for 8 min. The process comprised 30 cycles. The PCR program for *CYP2R1* exon 4 started at a hot temperature of 95 °C for 10 min, followed by denaturation at 95 °C for 30 s, annealing at 60 °C for 50 s, extension at 72 °C for 30 s, and final extension at 72 °C for 8 min. This reaction was repeated 30 times. The PCR program of the *GC* exon 8 was also started at a hot temperature of 95 °C for 10 min, followed by denaturation at 95 °C for 30 s, annealing at 49 °C for 50 s, extension at 72 °C for 30 s, and final extension at 72 °C for 8 min. This was repeated 30 times. PCR was performed for the case and control samples, and then the product sizes of exon 4 of the *CYP2R1* gene, exon 4 of the *CYP27B1* gene, and exon 8 of the *GC* gene were visualized on 2% agarose gel; these are given in the Supplementary Materials. 

A Gene JET PCR Purification-Kit, Catalog No FERK0721 (Thermo Fisher Scientific, Waltham, MA, USA), was used to purify the gene fragments. The PCR sequencing reactions were performed using a Brilliant Dye™ terminator (v3.1) cycle sequencing kit (NimaGen B.V. Hogelandseweg 88 NL 6545 AB, Nijmegen, The Netherlands). The PCR sequencing program for *CYP27B1*, *CYP2R1* exon 4, and *GC* exon 8 started at 96 °C for 1 min, followed by denaturation at 96 °C for 10 s, annealing at 50 °C for 5 s, and extension at 60 °C for 4 min. This reaction was repeated 25 times. The reagents used in PCR sequencing were 2 µL of sequencing dye, 2 µL of sequencing buffer, 0.4 µL of primers, and 2 µL of purified product. The purified products were sequenced using an Applied Biosystems® 850 Lincoln Centre Drive, Foster City, CA 94404, USA.

### 2.5. In Silico Modeling of GC and CYP2R1 Protein and Binding Pocket Analysis of Vitamin D3

The protein Sequences of *GC* and *CYP2R1* were downloaded from Ensembl (www.ensembl.org, accessed on 20 March 2024) [28]. In the case of *CYP2R1*, the transcript ID selected was ENST00000334636.10, which has 2214 base pairs and 501 amino acids. The accession number of the consensus coding sequence (CCDS) was CCDS7818, the UniProt Match was Q6VVX0, and the RefSeq Match was NM_024514.5. In the *GC* gene, the transcript ID selected was ENST00000273951.13, which has 1685 base pairs and 474 amino acids. The accession number of CCDS was CCDS3550, the UniProt Match was P02774, and the RefSeq Match was NM_000583.4. The Swiss-Model Suite [29] was used to generate models of *GC* and *CYP2R1* based on homology templates (PDB ID: 1j78 and 3c6g) for wild and mutated proteins. The vitamin D3 (cholecalciferol) coordinate file and SMILES were retrieved from PubChem [30] and used as a ligand. The modeled target proteins and ligand were uploaded to SWISS-DOCK [31] for protein–ligand docking. The binding pocket analysis of wild and mutated proteins was performed using online biological software Discovery Studio Visualizer (https://discover.3ds.com/discovery-studio-visualizer-download, accessed on 20 March 2024) and PyMol (https://www.pymol.org/, accessed on 20 March 2024) [32]. We also retrieved 1j78 and 3c6g protein complexes with vitamin D3 from RCSB [33] and used them as references for the pocket binding analysis. These polymorphisms were analyzed using the Mutation Taster [34] and SIFT [35] to determine the pathological effect of mutations on the structure of proteins.

### 2.6. Statistical Analysis

The statistical analysis of all polymorphisms was performed by SNPstat [36] R Studio (2025.05.1 Build 513), and SPSS 23 software. A logistic regression test adjusted by gender and age was conducted to calculate odds ratios (ORs) and 95% confidence intervals (CIs), and the chi-square (χ^2^) and Fisher’s exact tests were used to acquire allelic and genotypic frequencies, Hardy–Weinberg equilibrium (HWE), and associations. A *p*-value of less than 0.05 was considered statistically significant. The AB1 sequencing files were interpreted using the software Finch TV version 1.4 and Bio Edit version 7.2 [37].

## 3. Results

### 3.1. Genotyping Analysis of rs7041 (GC), rs782153744, rs200183599 (CYP2R1), rs118204011, and rs28934604 (CYP27B1 Gene)

The genotypic and allelic frequencies of *CYP2R1, CYP27B1*, and the *GC* genes are given in Table 1.

In the rs7041-dominant model, the minor alleles are absent in the control group, while half of the cases have one (G/A) allele. This shows a positive association between VDD and the minor allele (odds ratio: 1.935, 95% CI = 1.735–2.159 *p*-value < 0.001). The codominant model was significantly associated with VDD (*χ*^2^ = 191.209, *df* = 4, *p* < 0.001), and in Fisher’s exact test, *p* < 0.001. These results suggested that minor homozygotes (G/A) were present in the cases and absent in the controls.

The logistic regression analysis of the rs782153744-dominant model showed a *p*-value < 0.001, which indicated that the genotype has a sufficient contribution to enable its use in predicting disease. The individuals with G/C, G/T, and C/C genotypes had lower odds of having VDD (protective association) than those with the G/G genotype, which is the reference (odds ratio: 0.19, 95% confidence interval = 0.10–0.33, *p*-value: 3.09 *×* 10^−8^). For the recessive (OR: 0.19, 95% CI: 0.10–0.33, *p*-value < 0.001) and additive (OR: 0.15, 95% CI: 0.08–0.27, *p*-value < 0.001) genetic models, the logistic regression analysis also showed a protective association with VDD.

In rs200183599, the genotypic frequency of the AA, AG, and GG genotypes was the same in the case and control groups. Similarly, the frequency of the A and G alleles was also the same in the case and control groups. A 100% frequency of the A allele and AA genotype was observed in the cases and controls. In terms of genotypes, *p =* 1.00, OR 1.00, and CI; 0.019–50.64, and in terms of alleles, *p =* 1.00, OR 1.00, and CI; 0.019–50.52.

The rs118204011 and rs28934604 (*CYP27B1* gene) polymorphisms had an odds ratio of 1 and a *p*-value of 1, which shows no association of vitamin D deficiency with the rs118204011 and rs28934604 variants of the *CYP27B1* gene in all genetic models (Supplementary Materials).

### 3.2. Sanger Sequencing of Exons

Exon 4 of the *CYP2R1* gene and *CYP27B1* gene, and exon 8 of the *GC* gene, had the same electropherogram sequences in the cases and controls, with no variations found, as shown in Supplementary Materials.

### 3.3. In Silico Molecular Modeling and Binding Pocket Analysis of GC and CYP2R1 Proteins with Vitamin D3

**Figure 1:**
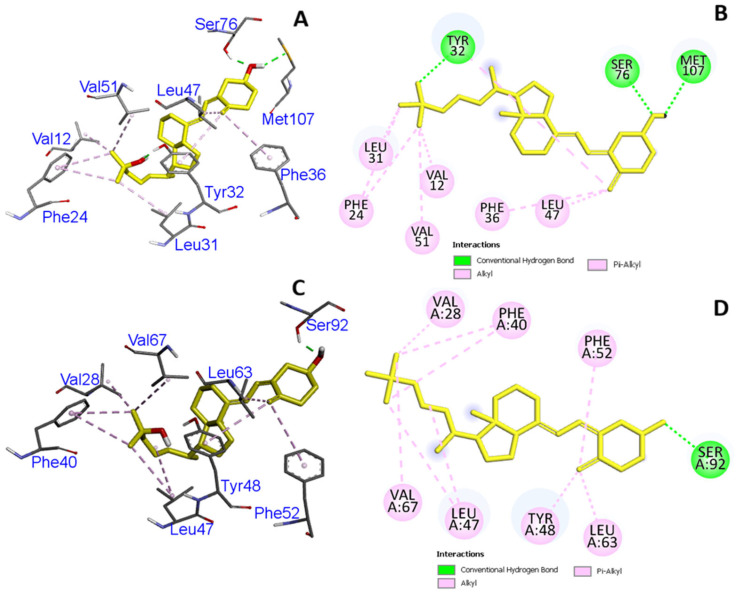
(**A**,**B**) Three- and two-dimensional representations of the binding interaction of the native *GC* protein with VD3. (**C**,**D**) Three- and two-dimensional representations of binding interaction of the mutated *GC* protein with VD3. Yellow color denotes vitamin D3 as a ligand.

**Figure 2:**
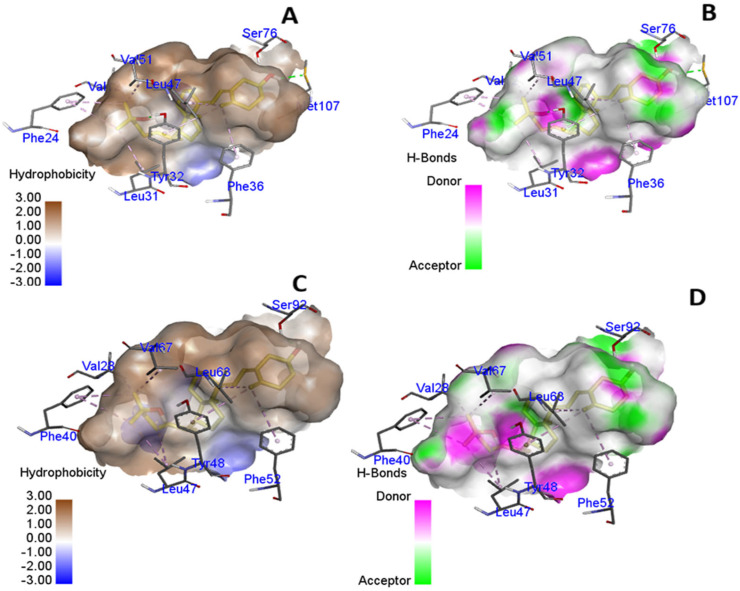
Hydrogen bonds and hydrophobicity in mutated *GC* (**A**,**B**) and native *GC* proteins (**C**,**D**).

The rs7041 polymorphism changes the amino acid aspartic acid to glutamic acid at position 432 in the *GC* protein (D432E), and the rs782153744 polymorphism changes proline to serine, alanine, and threonine at position 376 (P376S, P376A, P376Thr). We observed changes in the binding pocket of vitamin D3 inside the *GC* protein. The rs7041 created changes in the binding interaction site and added new interacting amino acid (glutamic acid) residues in the mutated state. A 2D structure was also built (Figure 1A–D), and we found that Leu12 was retained in a mutated state in the binding interaction, while new interacting amino acids (Phe52, Try48, Phe40, Val67, Leu63, Val28, and Phe40) were formed in the binding pocket. We analyzed the hydrogen bonding and hydrophobicity of native and mutated *GC* protein and found differences in these interacting forces (Figure 2A–D).

**Figure 3:**
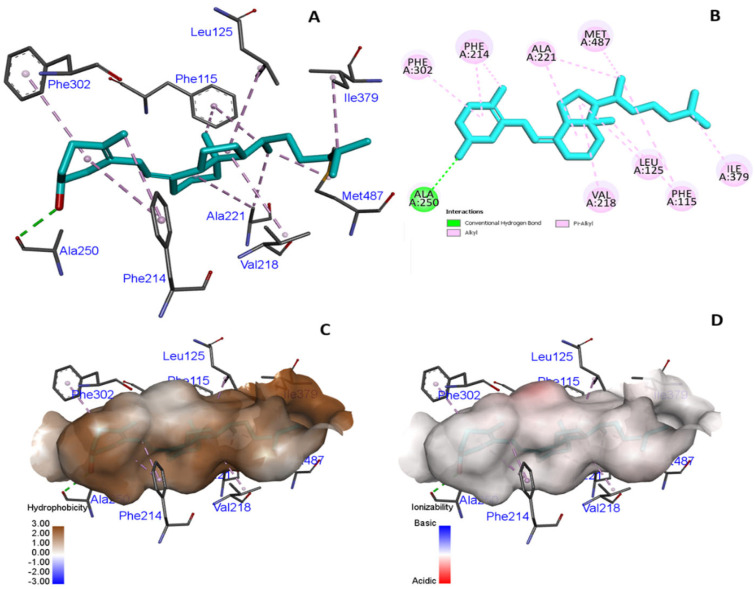
(**A**–**D**) Native *CYP2R1* protein showing interactive residue with VD3 binding pocket, hydrophobicity, and ionizability.

**Figure 4:**
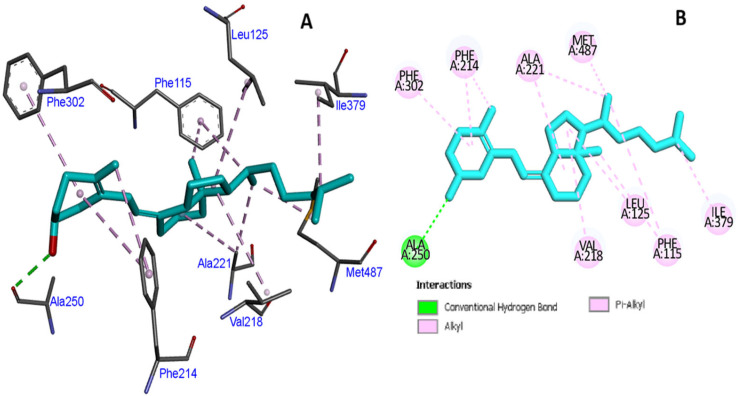
Mutated *CYP2R1* protein at position 376 with a serine residue complex with VD3 showing binding interaction (**A**) and two-dimensional schematic representation (**B**).

**Figure 5:**
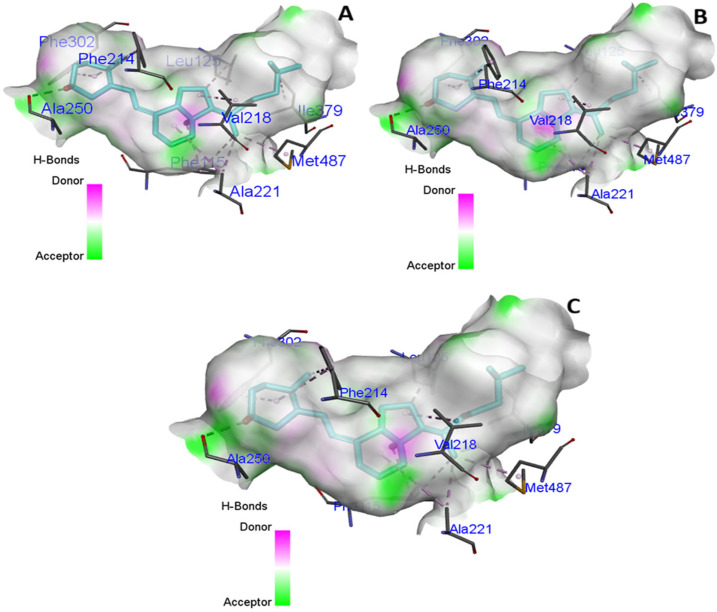
Hydrogen binding pocket analysis of *CYP2R1* protein. (**A**) Native protein with proline at position 376. (**B**,**C**) Mutated *CYP2R1* protein with serine and alanine at position 376.

The docking of *CYP2R1* with VD3 in the native state, compared to the mutated state, did not result in a change in binding interactions (Figure 3 and Figure 4), including hydrogen bonding and hydrophobicity. We analyzed the hydrogen bonding of native protein carrying proline at position 376, and serine and alanine, in the mutated state and found no difference in hydrogen bonding state (Figure 5).

The Supplementary Materials show a Ramachandran plot and the Molprob scores of our models.

## 4. Discussion

The rs7041 (A/C/G/T) is a missense variant located on exon 12 in the *GC* gene. Our variants showed a higher minor allele (G, A) frequency in case subjects than in control subjects. The G and A alleles in both rs7041 polymorphisms (T > G, T > A) were not present in the control group and were only observed in the vitamin D-deficient group. Therefore, the G and A alleles were identified as risk factors for VDD, with *p*-values of less than 0.001. In our study, the controls had only the TT genotype (dominant model—odds ratio: 1.935, 95% CI = 1.735–2.159, *p*-value *<* 0.001; codominant model—*χ*^2^ = 191.209, *df* = 4, *p* < 0.001). Our results are supported by a British study that reported that minor alleles of *GC rs7041* were associated with low serum vitamin D levels across the whole year in children residing at northern latitudes. This is probably due to altered serum vitamin D reactions to sunlight exposure, as identified with (*GC*) rs7041 [38]. Another study echoed these findings, finding that celiac disease patients with the rs7041 GG genotype and G allele both had a *p*-value of 0.03, and their serum vitamin D levels were low. This shows a statistically significant association between these variants and vitamin D deficiency [39]. In addition, the genetic variant rs7041 (A > C) of the *GC* gene has been identified as a risk factor in autism spectrum disorder. It was found that children who had minor allele C and vitamin D insufficiency were more likely to have autism spectrum disorder, with an odds ratio of 3.14 and a 95% confidence interval of 1.36–7.27, compared to those with reference allele A and sufficient vitamin D levels [40]. These studies support our results, which identified minor alleles (G, A) as risk alleles associated with low vitamin D levels.

An Iranian study recruited 196 healthy subjects and genotyped rs7041 with restriction fragment length polymorphism (RFLP). It reported that TT and GT genotypes were observed significantly more frequently in vitamin D-deficient and -insufficient groups. The frequency of the GT genotype in the deficient group was 31.3%, and in the insufficient group, it was 52.1% (with a *p*-value of 0.02). The TT genotype frequency was 33.3% in the vitamin D-deficient group and 13.7% in insufficient subjects [41]. Another study showed that the SNP rs4588 (*GC*) was associated with decreased vitamin D levels, and that rs7041 plays a protective role. The mutant allele (G) of rs7041 has a greater capacity to increase survival in cirrhosis patients with or without hepatocellular carcinoma compared with the TT wild genotype [42]. These studies contradict our results, which showed the TT genotype in the controls only. The G allele is associated with low vitamin D levels. From this perspective, the different factors that are involved in the pathophysiology of vitamin D deficiency must also be taken into account. The post hoc power analysis in rs7041 identified >99% power to detect effect sizes (Cramer’s V = 0.565) within the sample (300 cases, 300 controls).

In the molecular modeling and ligand-binding pocket analysis, we observed a notable alteration in the VD3 binding pocket in the mutated *GC* protein. While aspartic and glutamic acids are both acidic residues, they differ structurally, as aspartic acid has a shorter side chain (–CH_2_–COO^−^) and glutamic acid has a longer side chain (–CH_2_–CH_2_–COO^−^). These extensions can cause steric hindrance in tight binding pockets, alteration in electrostatic surface topology, displacement or rotation of nearby residues, and disruption or formation of hydrogen bonds or salt bridges. Molecular modeling (Discovery Studio) showed the clear addition of binding residue of the vitamin D3 binding pocket in the *GC* mutant protein. Verboven [43], for the first time, used the crystal structure of *GC* in complex with VD3, and we used this information to confirm our modeling. This change in amino acid likely reshaped the pocket geometry, influencing ligand orientation or binding stability. The methylene group might have changed the side chain length and rotamer preferences, and the observed change in binding site residues may have reduced the binding affinity for 25(OH)D_3_ or 1,25(OH)_2_D_3_; altered ligand orientation, affecting downstream transport and cellular uptake; and influenced thermodynamic stability and protein–ligand interaction energy. However, we recommend further molecular dynamics simulations. The statistical significance of our results and evidence of structural impact indicate that the rs7041 polymorphism is a biologically valid threat associated with VDD.

The SNP rs782153744 is a missense variant containing G/A/C alleles in the *CYP2R1* gene. This polymorphism is located in exon 4 of the *CYP2R1* gene on chromosome 11, and a change in codon can change proline to serine, alanine, or threonine in the 25-hydroxylase protein. The rs782153744 showed a protective association in the additive (OR: 0.15, 95% CI: 0.08–0.27, *p*-value< 0.001), recessive (OR: 0.19, 95% CI: 0.10–0.33, *p*-value < 0.001), and dominant (OR: 0.19, CI = 0.10–0.33, *p*-value < 0.001) models. In rs782153744, the genetic models have *p*-values of less than 0.001 which shows that these genotypes can predict disease. The subjects with C/C, G/T, and G/C genotypes had lower odds of acquiring VDD (protective role) than those with the reference G/G genotype. There are no studies about rs782153744, and the Single-Nucleotide Polymorphism Database has also reported no publications about this polymorphism. Thus, this pilot study was conducted in apparently healthy members of the population at the national and international levels, and demonstrated the protective association of this polymorphism in vitamin D-deficient individuals. This pilot study has an adequate sample size and a strong effect size, as GG frequency was 19.3% in the cases and 4.7% in the controls. In addition, the frequency of G/C was 62% in the cases and 92.3% in the controls, indicating that it is protective. This population-based study of protective polymorphisms may indicate their potential for involvement in specific interventions and pharmacogenomics. The identification of protective alleles (G, T, C) is useful for stratifying populations that have low susceptibility to vitamin D deficiency, and will be useful for informing screening approaches and supplement prescriptions. The genetic screening of rs7041 and rs782153744 polymorphisms will allow physicians to better evaluate VDD risk, design targeted approaches to prevent vitamin D deficiency, and maintain optimum vitamin D levels to attain comprehensive health models. The Bonferroni-adjusted threshold was α/n = 0.05/7 = 0.0071, and rs7041 and the SNP rs782153744 had *p*-values of less than 0.001, which remained significant after Bonferroni correction.

The in silico modeling (substitution of proline with serine) of wild p53 protein showed cavity formation in the hydrophobic region, the presence of a thermodynamically unfavorable polar group, the presence of the hydroxyl (OH) oxygen atom of serine, and a decrease in van der Waals forces, which are likely to produce alterations in local architecture [44]. The in silico modeling showed that the most common protein variations were related to hydrophobicity and the sizes of amino acids. The substitutions of glycine and proline were mostly placed in the pathogenic category [45]; though we did not observe a change in the binding pocket geometry of VD3 with *CYP2R1*. Proline was likely not a direct component of the pocket’s shape or chemical environment. Replacement with serine or alanine did not significantly distort the 3D conformation or alter the positioning of critical residues for VD3 binding. Although serine is more flexible and capable of forming H-bonds, the stability of the local backbone conformation might have been due to nearby structurally rigid residues and stabilizing secondary structures (e.g., helix or sheet constraints). This genetic variant might be in linkage disequilibrium with another polymorphism or influence gene regulatory mechanisms that do not modify protein structure.

A study observed the effect of coding variation in the 25-hydroxylase enzyme, and found that 21 single-nucleotide polymorphisms, including rs200183599, modified vitamin D homeostasis by reducing enzyme activity (<25% of wild-type enzymes), and two variants increased 25-hydroxylase enzyme activity (>175% of wild-type enzymes), while 21 polymorphisms exhibited regular activity. This was due to a change in amino acids affecting protein interactions. These variations reflect variations based on geographic distribution to adapt to regional sunlight exposure and maintain vitamin D homeostasis [46]. The results of this study were contradictory to our findings, which showed a *p*-value of 1 and an OR of 1. In terms of genotypes CI: 0.019–50.64, and in terms of alleles, CI: 0.019–50.52. The wide range of confidence intervals augments our SNP genotyping result, which shows only one allele variant in the population (cases and controls). This monomorphic outcome shows that rs200183599 likely has no association with vitamin D deficiency in our population. This is an informative and common result in genetic pilot studies. This directs us to select other genetic variants to observe the effect of VDD in our population. However, our study is supported by research conducted in Saudi Arabia, which showed that the rs12794714 (*CYP2R1*) variant in not associated with decreased levels of vitamin D. The *p*-values of both genotypes of rs12794714 (GA and AA) were statistically insignificant (0.29 and 0.58, respectively) [47]. This correlated with a study conducted in China to determine the triangular relation between *CYP2R1* polymorphisms (rs1993116 and rs10766197), VDD, and type 2 diabetes mellitus. It found a significant relationship between these polymorphisms and the risk of T2DM. Neither polymorphism had an association with serum vitamin D levels [48]. These findings are in agreement with our results, which showed no association of rs200183599 with VDD; it was equally distributed in healthy and vitamin D-deficient subjects.

The *CYP27B1* polymorphism (rs118204011 (C/T) has reference allele “C” and minor allele “T”. A genotypic analysis between pulmonary tuberculosis (TB) patients and healthy controls showed no statistically significant association, with the mutant genotype TT completely absent in this population, and rs118204012 (AA genotype) was associated with TB susceptibility and vitamin D insufficiency [49]. Our research also showed no association of rs118204011 with low vitamin D levels, with a *p*-value of 1 and an OR of 1. Many studies have shown a significant association of the rs118204011 polymorphism with VDD. The *CYP27B1* polymorphism rs118204011 affects serum vitamin D levels and modulates immune response, and is associated with multiple sclerosis and low vitamin D levels. This study concluded that a rare variant (rs118204011) in the *CYP27B1* gene affects vitamin D activity and may increase susceptibility to developing multiple sclerosis [50]. An Iranian study also reported a significant association of the *CYP27B1* polymorphism (*rs4646536*) with vitamin D insufficiency (*p*-value < 0.0005; OR = 4) [51]. The *CYP27B1* polymorphism considerably changes alpha 1 hydroxylase enzyme activity in colon cancer cells; in addition, rs28934604, rs5891567, rs13377933, and rs2229103 decrease alpha 1 hydroxylase enzyme activity in cancer cells, while rs8176344 increases it [52]. In our study, rs28934604 was not found to be associated with VDD. The current study showed that genotypes of none of the studied genetic polymorphisms followed the Hardy–Weinberg equilibrium (*p* < 0.05). This may be due to the heterogeneous nature of the Pakistani population, which is multicultural, with inter-caste and consanguineous marriage practices, decreased allele segregation, random reproduction, and genetic recombination.

The high prevalence of VDD in this population is also reported to be due to certain extrinsic factors. An analysis revealed that around 70% of the population has VDD, with the average vitamin D levels in deficient individuals being about 18 nmol/L (minimum threshold = 20 nmol/L). This demonstrated that there is less intake of vitamin D in the Pakistani population [53]. According to the World Health Organization, the yearly average amount of particulate matter 2.5 (PM2.5) in Pakistan is 52.1 µg/m^3^, and it is ranked as the third-most polluted nation [54]. The subjects who lived in highly contaminated areas were vitamin D-deficient or had a higher risk of VDD [55]. In addition, dark skin and garments that cover the whole body except the face are also contributing factors.

Polymorphisms are population-based [56,57,58], which has led to revisions of the recommended daily allowance (RDA) and fortification of food items. It has also motivated researchers to develop vitamin D supplements that increase bioavailability in polymorphism-affected subjects. This research adds to the global gene pool data regarding the Pakistani population.

## 5. Conclusions

This study enhances our understanding of the role of rs782153744 (*CYP2R1*) as a novel protective polymorphism and highlights structural alterations in *GC rs7041* that may impact vitamin D3-binding ability. It also expands our understanding of the genetic influences of VDD in Pakistani (South Asian) populations. The rs200183599, rs118204011, and rs28934604 polymorphisms might not be associated with VDD in apparently healthy members of Pakistan’s population.

Limitations and Recommendations: Selected polymorphisms in each gene were studied due to limited financial resources, and the general population was stratified into cases and controls. We recommend exploring rs7041 on a large scale to obtain logical insight into this polymorphism with VDD.

## Figures and Tables

**Table 1:** Genotypic and allelic frequencies of various studied polymorphisms.

SNPs	Genotype and Alleles	Cases (n = 300)	Controls (n = 300)
rs200183599 (*CYP2R1*)	AA	300 (100%)	300 (100%)
	AG	0 (0%)	0 (0%)
	GG	0 (0%)	0 (0%)
	A	600 (100%)	600 (100%)
	G	0 (0%)	0 (0%)
rs782153744 (G > T) (*CYP2R1*)	GG	228 (76%)	289 (96%)
	GT	0 (0%)	0 (0%)
	TT	72 (24%)	11 (4%)
	G	456 (76%)	289 (96%)
	T	144 (24%)	11 (4%)
rs782153744 (G > C) (*CYP2R1*)	GG	84 (28%)	15 (5%)
	GC	1 (0.3%)	1 (0.3%)
	CC	215 (71.6%)	284 (94.6%)
	G	169 (28.16%)	31 (5.16%)
	C	431 (71.83%)	569 (94.83%)
rs28934604 (*CYP27B1*)	GG	259 (86%)	258 (86%)
	GA	6 (2%)	0 (0%)
	AA	35 (11.66%)	42 (14%)
	G	524 (87%)	516 (86%)
	A	76 (13%)	84 (14%)
rs118204011 (*CYP27B1*)	CC	215 (71.66%)	203 (67.66%)
	CT	0 (0%)	2 (0.6%)
	TT	85 (28.33%)	95 (31.66%)
	C	430 (71.66%)	408 (68%)
	T	170 (28.33%)	192 (32%)
rs7041 (T > G) (*GC*)	TT	191 (64%)	300 (100%)
	TG	2 (0.6%)	0 (0%)
	GG	107 (36%)	0 (0%)
	T	384 (64%)	600 (100%)
	G	216 (36%)	0 (0%)
rs7041 (T > A) (*GC*)	TT	239 (79.66%)	300 (100%)
	TA	1 (0.3%)	0 (0%)
	AA	60 (20%)	0 (0%)
	T	479 (79.83%)	600 (100%)
	A	121 (20.16%)	0 (0%)

## Data Availability

The data are contained in the article and Supplementary File.

## Supplementary Materials

The following supporting information can be downloaded at: https://www.mdpi.com/article/10.3390/cimb47100849/s1, Supplementary Materials are provided in the Supplementary File [59].
