# Supplementary material for: Identification and Association of CYP2R1, CYP27B1, and GC Gene Polymorphisms with Vitamin D Deficiency in Apparently Healthy Population and in Silico Analysis of the Binding Pocket of Vitamin D3
Source: Curr Issues Mol Biol. 2025 Oct 15;47(10):849. doi: 10.3390/cimb47100849 (PMC12563223; doi:10.3390/cimb47100849)
Supplement: Supplementary file 1 [file cimb-47-00849-s001.zip › cimb-3812603-supplementary.pdf]

DNA Extraction

Phenol-chloroform method (Sambrook and Russell, 2006) was used for DNA extraction, which has the following steps.

Cell lysis

The blood sample (700µl) was taken in an Eppendorf tube and mixed with 300µl of lysis solution A. It was prepared in falcon tube by adding sucrose(0.32M)320 µl , Tris (10mM)100 µl, MgCl2 (5mM) 50 µl, SDS(4%)2 µl and Triton-X100 (1%)100 µl. After it, distilled water was added to make it 10 ml. Both were mixed by vortexing and incubated at room temperature for 30 minutes. The eppendorf tube was then centrifuged at 13000xg for 15 minutes, and the pellet was taken. It was followed by 100 µl lysis solution B. It was prepared in a Falcon tube by the addition of Tris 100 µl, EDTA 40 µl, and NaCl 4 ml. At the end, distilled water was added to make it 10ml. After it, 4 µl proteinase K was added. The solution was then mixed by vortexing, and the eppendorf tube was incubated overnight at 37˚C.

Precipitation

In this step, the eppendorf tube was removed from 37˚C. The Phenol Chloroform Isoamyl alcohol solution (25; 24; 1) was shaken vigorously, and an equal volume was added to the eppendorf tube. It was vortexed for 2 minutes to get it mixed properly. The eppendorf tube was centrifuged for 15 minutes at 13000xg. The upper aqueous layer was then transferred to another labelled eppendorf tube. Then an equal volume of Chloroform was added, and this was followed by vortexing. The eppendorf tube was centrifuged at 13000xg for 10 minutes. The upper aqueous layer was then transferred to another labelled eppendorf tube. Then an equal volume of isopropanol (100%) was added to it. This was followed by 0.1% volume of 3M Na acetate. Finally, the eppendorf tube was placed at -20˚C for 1 hour.

Purification

The eppendorf tube was centrifuged at 13000xg for 15 minutes. The supernatant was discarded. The eppendorf tube was opened and placed in a vacuum concentrator for 30 minutes. The pellet get dried. It was followed by the addition of 100 µl of distil water. Vortexing was done to homogenize the solution. Finally, it was stored at -20^{0}C. A total of 600 samples were used to extract DNA manually.

Qualitative Determination of Extracted DNA

The agarose gel electrophoresis technique was used to analyse DNA quantitatively and qualitatively. The buffer 5X Tris-Borate-EDTA (TBE) was prepared. Its components included boric acid 27.5g, Trisma base 54g and EDTA 4.65g. These ingredients were dissolved in 1000 ml distilled water by using a hot plate and magnetic stirrer. The solution 5X TBE was then diluted to 1X TBE. This process was completed by adding 200ml of 5X TBE into 800 ml of distil water. This 1X TBE, was used to prepare 1% agarose gel. For preparation of 1% agarose gel, 40ml 1X TBE was measured

in 50ml cylinder and carefully poured into a beaker. Then 0.4g powdered agarose was measured in weight balance and placed in the beaker. It then positioned in the micro wave to dissolve agarose completely. Extreme care should be taken to prevent boiling of solution excessively. In the mean while rubber clamps were fixed on both sides of gel caste and combs were also placed at appropriate levels on both sides. Once the agarose was dissolved completely, then 4µl ethidium bromide (EtBr) dye was added to the heated agarose solution and mixed well. Then gel was poured into the gel caster and allowed it to solidify. It took 30-35 minutes. After solidification, the gel was placed in the gel electrophoresis tank, which had 1XTBE buffer at appropriate levels. Then 2 µl of DNA was mixed with 2 µl loading dye (6X), i.e., 0.25% bromophenol blue, and the samples were loaded in the wells. 1kb DNA ladder was added as a standard for confirmation of extracted DNA. The voltage was set at 80, and it took 40 minutes to assess DNA on the Gel Doc ™ XR+ Gel Documentation system (BioRad). The gel image was saved on this system.

All extracted DNA (300 controls and 300 cases) was observed by 2% agarose gel electrophoresis, and their images were saved in the Gel Doc system.

Table S1; Primers for *CYP2R1* rs200183599

Primer	Sequence 5’-3’direction
Forward inner	GCATTTTGTGATGCTTATTTAGATGATAC
Reverse inner	ATGATGGGTCATTTTTACCTTGATACA
Forward outer	CTCAGTCTTCTTGTATAATGCCTTTCC
Reverse outer	AACATGTATGAACCCTACATGTAAGGAT

Table S2; Primers for rs782153744 C>A

**Primer**	**Sequence** 5’-3’direction
Forward inner	TGAAGTTTTAAGATTCTGTAATATAGCTC
Reverse inner	GAGGTTGCATGGAAAATCCCTAACGT
Forward outer	TGGATGTATCTATATCCTATTGGCTTGT
Reverse outer	AAGTTCTCTTACCTAGGGAAAAAGGAAC

Table S3; Primers for rs782153744 G>C

**Primer**	**Sequence** 5'-3'direction
Forward inner	TGAAGTTTTAAGATTCTGTAATATAGATG
Reverse inner	AGAGGTTGCATGGAAAATCCCTAAAGG
Forward outer	CTGGATGTATCTATATCCTATTGGCTTG
Reverse outer	AAGTTCTCTTACCTAGGGAAAAAGGAAC

Table S4; Primer sequence of *CYP27B1* (rs28934604) G>A

**Primer**	**Sequence 5'-3'direction**
Forward inner primer	GAGGGACCCCGGCCCGATCA
Reverse inner primer	TCCAGGGCGAGAAGCTGCCGC
Forward outer primer	TCAAGCTGAAAGTCCCCGCCAGG
Reverse outer primer	TGGGAACCCCAAGATGCCCAATGG

Table S5; Primer sequence of *CYP27B1* (rs118204011*)* T>C

**Primer**	**Sequence 5'-3'direction**
Forward inner primer	CGGCACCCCAAGTCCAGACAGAAT
Reverse inner primer	TCAGGGCAGCTGTGATCTCTGAGTGTAG
Forward outer primer	CTGTTGCAGGGGATCCATTATGGCC
Reverse outer primer	TGGCCAGGAGTAGAGGGCCATTTTTC

Table S6; primers sequence of *GC* rs7041variant T>G

Primer	Sequence 5'-3'direction
Forward inner primer	GCAGAGCGACTAAAAGCAAAATTGCCTAAT
Reverse inner primer	GCTTTGCCAGTTCCGTGGGTGTGACC
Forward outer primer	GACTGGACTTCCAATTCAGCAGCGATTG
Reverse outer primer	AACACCAGGAAAAGCCTGTCACATAATGGC

Table S7; primers sequence of *GC* rs7041 variant T>A

Primer	Sequence5'-3'direction
Forward inner primer	GCAGAGCGACTAAAAGCAAAATTGCCTAAT
Reverse inner primer	AGCTTTGCCAGTTCCGTGGGTGTGACT
Forward outer primer	TGGACTTCCAATTCAGCAGCGATTTGT
Reverse outer primer	CACCAGGAAAAGCATGTCACATAATGGC

Tetra-ARMS PCR

This technique was used to genotype the single-nucleotide polymorphisms in target gene. It was a cost-effective and simple technique (Medrano and de Oliveira, 2014). In tetra-primer ARMS–PCR four primers are use in one PCR reaction to find out the genotypes. One set of inner primers and one set of outer primers are use to get amplification of the required gene. The outer primers serve as a template for two primers, which were allele-specific (inner primers) and then yields the allele-specific amplicon.

.

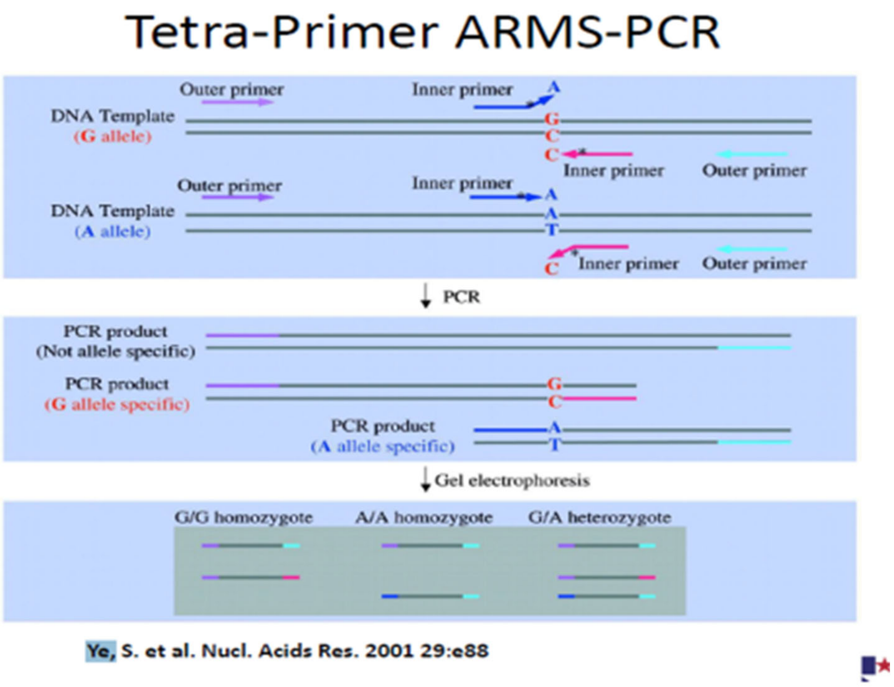

Medrano, R. F. V. & De Oliveira, C. A. 2014. Guidelines for the tetra-primer ARMS–PCR technique development. *Molecular biotechnology,* 56, 599-608.

Sambrook, J. & Russell, D. W. 2006. Purification of nucleic acids by extraction with phenol: chloroform. *Cold Spring Harbor Protocols,* 2006, pdb. prot4455.

Table S8; PCR Program for rs200183599 A> G polymorphism

Steps	Temperatures	Time	Repeats
Hot Start PCR	95°C	5minute	
Denaturation	95 °C	35seconds	
Annealing	60.4°C	25seconds	35 cycles
Extension	72 °C	25seconds	
Final Extension	72 °C	8 minutes	

Table S9; PCR reaction for rs200183599

	Ingredients	Quantity	Concentration
1.	Nuclease free water	11.7 µl	
2.	Taq buffer (Bio basic Canada}	2 µl	1xPCR
3.	25mM MgCl2(Bio basic Canada}	1.2 µl	1.5mm
4.	10mM dNTPs (dinucleotide triphosphates) Mix (Bio basic Canada}	0.4 µl	0.2mm
5.	Inner Forward (Thermo scientific)	0.8 µl	1P mol/ µl
6.	Inner Reverse	0.8 µl	1P mol/ µl
7.	Outer Forward	0.8 µl	1P mol/ µl
8.	Outer Reverse	0.8µl	1P mol/ µl
9.	Taq polymerase (Bio basic Canada}	0.5 µl	1 µl

Table S10; PCR Programme for rs782153744 G>T polymorphism

Steps	Temperatures	Time	Repeats
Hot Start PCR	95°C	5minute	
Denaturation	95 °C	35 seconds	
Annealing	65.2°C	1minute and 10second	35 cycles
Extension	72 °C	1 minute and 10 second	
Final Extension	72 °C	5 minutes	

Table S11; Reagents used in PCR for rs782153744 G>T polymorphism

Serial no.	Ingredients	Quantity	Concentration
1.	Nuclease free water	10.9 µl	
2.	Taq buffer (Bio basic Canada}	2 .5µl	1xPCR
3.	25mM MgCl2(Bio basic Canada}	2 µl	1.5mm
4.	10mM dNTPs Mix (Bio basic Canada}	0.5 µl	0.2mm
5.	Inner Forward(Thermo scientific)	0.5 µl	1P mol/ µl
6.	Inner Reverse	0.5 µl	1P mol/ µl
7.	Outer Forward	0.8 µl	1P mol/ µl
8.	Outer Reverse	0.8µl	1P mol/ µl
9.	Taq polymerase(Bio basic Canada}	0.5 µl	1 µl

Table S12; PCR Programme for rs782153744 G>C polymorphism

Steps	Temperatures	Time	Repeats
Hot Start PCR	95°C	5minute	
Denaturation	95 °C	35 seconds	
Annealing	65.2°C	1 minute and 10second	35 cycles
Extension	72 °C	1 minute and 10second	
Final Extension	72 °C	8 minutes	

Table S13; Reagents used in PCR for rs782153744 G>C polymorphism

Serial no.	Ingredients	Quantity	Concentration
1.	Nuclease free water	11.6 µl	
2.	Taq buffer (Bio basic Canada}	2 µl	1xPCR
3.	25mM MgCl2(Bio basic Canada}	1.5 µl	1.5mm
4.	10mM dNTPs (dinucleotide triphosphates) Mix (Bio basic Canada}	0.4 µl	0.2mm
5.	Inner Forward(Thermo scientific)	0.6 µl	1P mol/ µl
6.	Inner Reverse	0.6 µl	1P mol/ µl
7.	Outer Forward	0.9 µl	1P mol/ µl
8.	Outer Reverse	0.9µl	1P mol/ µl
9.	Taq polymerase(Bio basic Canada}	0.5 µl	1 µl

Table S14: PCR Program for rs 118204011T>C

Steps	Temperatures	Time	Repeats
Hot Start PCR	95°C	5minute	
Denaturation	95 °C	35seconds	
Annealing	69°C	35seconds	35 cycles
Extension	72 °C	35seconds	
Final Extension	72 °C	8 minutes	

Table S15; PCR reaction for rs118204011T>C

	Ingredients	Quantity	Concentration
1.	Nuclease free water	11.2 µl	
2.	Taq buffer (Bio basic Canada}	2 µl	1xPCR
3.	25mM MgCl2(Bio basic Canada}	1.5 µl	1.5mm
4.	10mM dNTPs (dinucleotide triphosphates) Mix (Bio basic Canada}	0.4 µl	0.2mm
5.	Inner Forward (Thermo scientific)	0.9 µl	1P mol/ µl
6.	Inner Reverse	0.9µl	1P mol/ µl
7.	Outer Forward	0.8 µl	1P mol/ µl
8.	Outer Reverse	0.8µl	1P mol/ µl
9.	Taq polymerase (Bio basic Canada}	0.5 µl	1 µl

Table S16; PCR Programme for rs28934604G>A

Steps	Temperatures	Time	Repeats
Hot Start PCR	95°C	5minute	
Denaturation	95 °C	35seconds	
Annealing	74°C	35seconds	35 cycles
Extension	72 °C	35seconds	
Final Extension	72 °C	8 minutes	

Table S17; PCR reaction for rs28934604

	Ingredients	Quantity	Concentration
1.	Nuclease free water	11.7 µl	
2.	Taq buffer (Bio basic Canada}	2 µl	1xPCR
3.	25mM MgCl2(Bio basic Canada}	1.2 µl	1.5mm
4.	10mM dNTPs (dinucleotide triphosphates) Mix (Bio basic Canada}	0.4 µl	0.2mm
5.	Inner Forward(Thermo scientific)	0.8 µl	1P mol/ µl
6.	Inner Reverse	0.8 µl	1P mol/ µl
7.	Outer Forward	0.8 µl	1P mol/ µl
8.	Outer Reverse	0.8µl	1P mol/ µl
9.	Taq polymerase(Bio basic Canada}	0.5 µl	1 µl

Table S18; PCR Programme for *GC* rs7041 T>G polymorphism

Steps	Temperatures	Time	Repeats
Hot Start PCR	95°C	5minute	
Denaturation	95 °C	40 seconds	
Annealing	69°C	1minute	35 cycles
Extension	72 °C	1 minute	
Final Extension	72 °C	8 minutes	

Table S19; Reagents used in PCR for *GC* rs7041 T>G polymorphism

Serial no.	Ingredients	Quantity	Concentration
1.	Nuclease free water	11.5 µl	
2.	Taq buffer (Bio basic Canada}	2 µl	1xPCR
3.	25mM MgCl2(Bio basic Canada}	1.5 µl	1.5mm
4.	10mM dNTPs (dinucleotide triphosphates) Mix (Bio basic Canada}	0.4 µl	0.2mm
5.	Inner Forward(Thermo scientific)	0.6 µl	1P mol/ µl
6.	Inner Reverse	0.6 µl	1P mol/ µl
7.	Outer Forward	0.8 µl	1P mol/ µl
8.	Outer Reverse	0.8µl	1P mol/ µl
9.	Taq polymerase(Bio basic Canada}	0.5 µl	1 µl

Table S20; PCR Programme for *GC* rs7041 T>A polymorphism

Steps	Temperatures	Time	Repeats
Hot Start PCR	95°C	5minute	
Denaturation	95 °C	40 seconds	
Annealing	69°C	1 minute	35 cycles
Extension	72 °C	1 minute	
Final Extension	72 °C	8 minutes	

Table S21; Reagents used in PCR for *GC* rs7041 T>A polymorphism

Serial no.	Ingredients	Quantity	Concentration
1.	Nuclease free water	11.5 µl	
2.	Taq buffer (Bio basic Canada}	2 µl	1xPCR
3.	25mM MgCl2(Bio basic Canada}	1.5 µl	1.5mm
4.	10mM dNTPs (dinucleotide triphosphates) Mix (Bio basic Canada}	0.4 µl	0.2mm
5.	Inner Forward(Thermo scientific)	0.8 µl	1P mol/ µl
6.	Inner Reverse	0.8 µl	1P mol/ µl
7.	Outer Forward	0.6 µl	1P mol/ µl
8.	Outer Reverse	0.6µl	1P mol/ µl
9.	Taq polymerase(Bio basic Canada}	0.5 µl	1 µl

Table S22: Multiple logistic regression test for **rs118204011** adjusted with age, gender

**Model**	**Genotype**	**Cases**	**Control**	**OR(95%CI)**	***p*-value**	**AIC**	**BIC**
Recessive	C/C-CT	215(71.7%)	205(67.7%)	1.00	1	396	1266.6
	T/T	85(28.3%)	95(31.7%)				
Codominant	C/C	215(71.7%)	203(67.7%)	1.00	1	398	1273
	C/T	0(0%)	2(0.7%)				
	T/T	85(28.3%)	95(31.7%)				
Dominant	C/C	215(71.7%)	203(67.7%)	1.00	1	396	1266.6
	C/T- T/T	85(28.3%)	97(32.3%)				
Over dominant	C/C- T/T	300(100%)	298(99.3%)	1.00	1	396	1266.6
	CT	0(0%)	2(0.7%)				

Table S23: Multiple logistic regression analysis for **rs28934604** adjusted with age and gender

**Model**	**Genotype**	**Cases**	**Control**	**OR(95%CI)**	***P*- value**	**AIC**	**BIC**
Recessive	G/G-G/A	265(88.3%)	258(86%)	1.00	1	12	38.4
	A/A	35(11.7%)	42(14%)				
Co dominant	G/G	259(86.3%)	258(86%)	1.00	1	14	44.8
	G/A	6(2%)	0(0%)				
	A/A	35(11.7%)	42(14%)				
Dominant	G/G	259(86.3%)	258(86%)	1.00	1	12	38.4
	G/A- A/A	41(13.7%)	42(14%)				

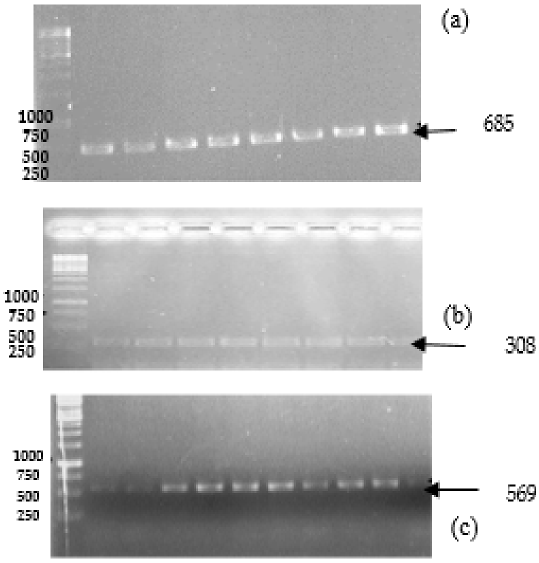

Figure S1; a; The exon 4 of *CYP2R1* gene product size 685bp, b; exon 4 of *CYP27B1*gene product size 308bp c; exon 8 of *GC* gene product size 569bp visualized on 2% agarose gel

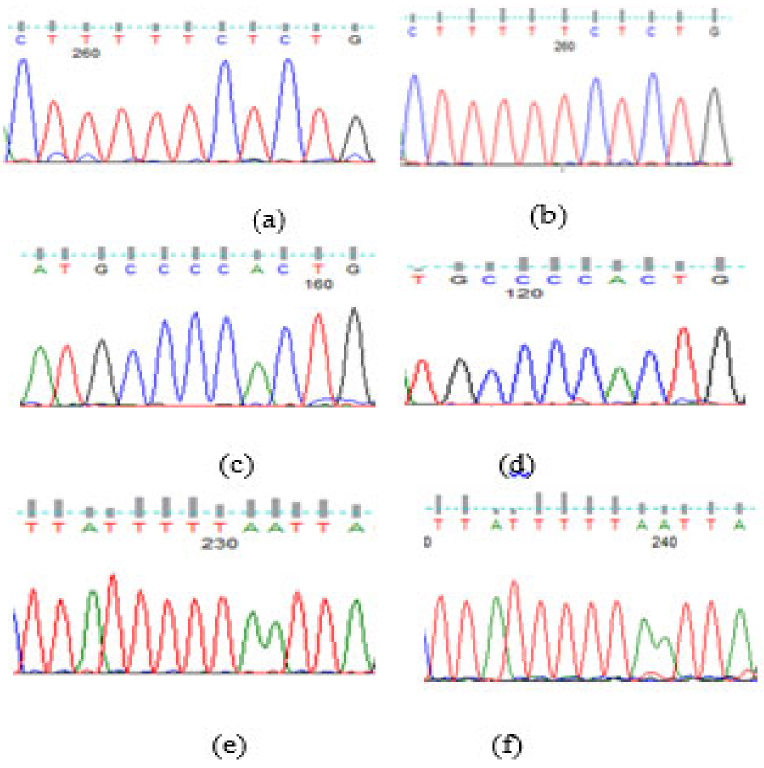

Figure S2**:** The electropherogram sequences of exon 4 *CYP2R1,* exon 4 *CYP27B1* gene (a) *(*b), exon 4 *CYP27B1* gene (c) (d), and exon 8 *GC* gene (e) (f) in cases and controls. Red=T Thymine, Blue= C Cytosine, Black=G Guanine. Green=A Adenine

Table S24: Ramachandran features of *GC* protein

**1. Ramachandran features**	**Wild *GC* Protein**	**Mutant *GC* Protein**
molprobity score	1.15	1.15
clash score	1.00	1.00
Ramachandran favored	98.01%	98.01%
Ramachandran outliers	0.22%,	0.22%
rotamer outliers	2.91%,	2.91%
C-Beta Deviations	2%	2%
bad bonds	1 / 3626	1 / 3627
bad angles	29/4916	28 / 4917
2. QMEAN Z-Scores		
QMEAN	0.13	- 0.14
Cβ	2.43	2.46
All atom	4.28	4.28
Solvation	2.32	2.35
Torsion	1.50.	-1.52
3.GMQE	0.94	
4. QMEANDisco Global	0.91 ±0.5	

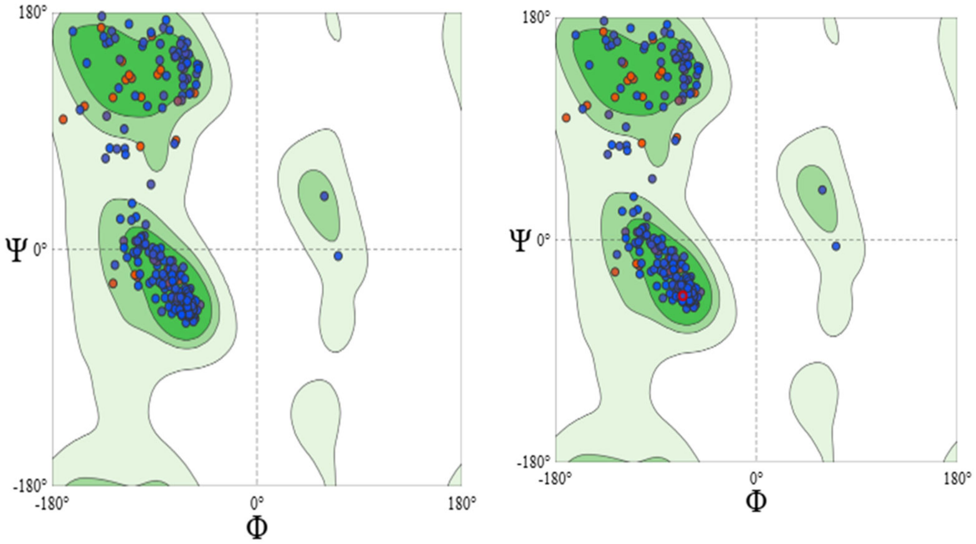

2a (Native *GC*)

2b (Mutated *GC*)

Figure S3; shows the Ramachandran plot, which has secondary structures like parallel and anti-parallel beta sheets, right-handed alpha helix, left-handed alpha helix, and right-twisted beta sheets of GC models.

Table S25: Ramachandran features of the *CYP2R1* protein

**1. Ramachandran features**	**Wild *CYP2R1* Protein**	**Mutant *CYP2R1* Protein**
molprobity score	0.50	0.50
clash score	0.00	0.00
Ramachandran favored	98.60%,	98.40%
Ramachandran outliers	0%	0%
rotamer outliers	0.69%	0.69%
C-Beta Deviations	0%,	0%
bad bonds	0 / 4169	0%
bad angles	18 / 5646	20 / 5642
2. QMEAN Z-Scores		
QMEAN	0.36	- 0.26
Cβ	0.63	0.61
All atom	0.68	0.61
Solvation	1.00	0.98
Torsion	0.13	0.04
3.GMQE	0.92	
4. QMEANDisco Global	0.94±0.5	

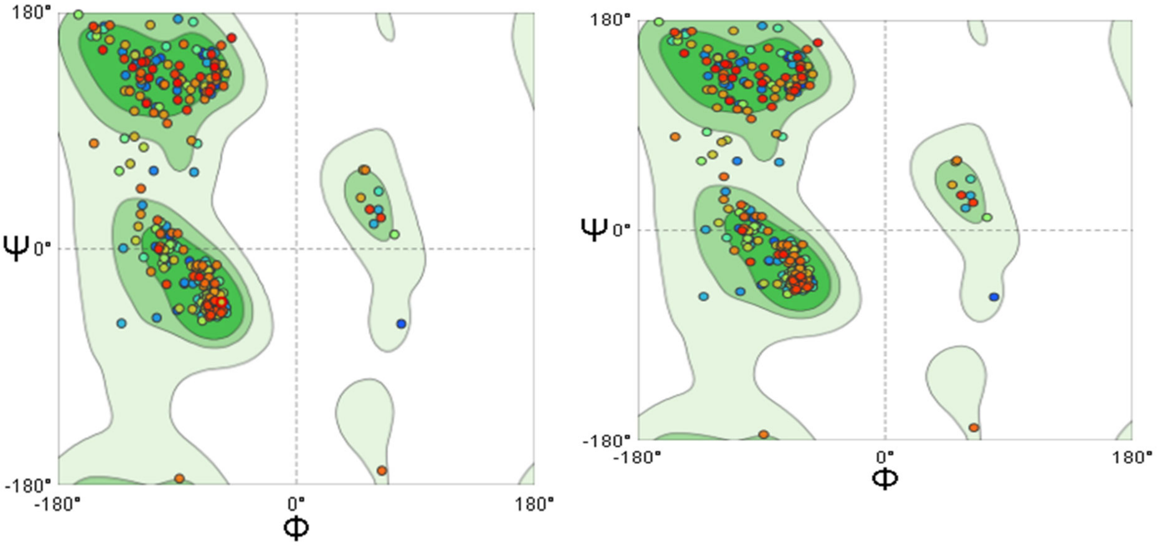

3a (native *CYP2R1*)

3b (Mutated *CYP2R1*)

Figure S4; shows the Ramachandran plot, which has secondary structures like parallel and anti-parallel beta sheets, right-handed alpha helix, left-handed alpha helix, and right-twisted beta sheets of GC models.

Army Medical College
Abid Majeed Road
Rawalpindi Cantt
Tel #: 051-5516755
Ext - 330
ERC / ID / 80
Dated 30 Nov 2020

CERTIFICATE

The Ethical Review Committee of Army Medical College (AMC) has evaluated the ethical aspects of the research Synopsis **"Identification and Computational Modeling of Genetic Polymorphisms in CYP27B1, CYP2R1 and GC Genes Associated with Vitamin D_3 Deficiency in Pakistan"** and accorded approval to be conducted from Jan 2018 to Jan 2025.

Lt Col
Secretary Ethical Review Committee
(Mudassar Noor)

Lt Col
Secretary Ethical Review Committee
Army Medical College
(Mudassar Noor)

COUNTERSIGNED

Maj Gen
President Ethical Review Committee
Army Medical College
(Sayed Nusrat Raza)

Maj Gen
President Ethical Review Committee
(Sayed Nusrat Raza)

Dated: 30 Nov 2020

To : Biochemistry Dept
Info: Saima Manzoor
